# *Chromobacterium violaceum* Septicaemia and Urinary Tract Infection: Case Reports from a Tertiary Care Hospital in South India

**DOI:** 10.1155/2016/6795743

**Published:** 2016-09-22

**Authors:** Vishnu Kaniyarakkal, Shabana Orvankundil, Saradadevi Karunakaran Lalitha, Raji Thazhethekandi, Jahana Thottathil

**Affiliations:** Government Medical College Kozhikode, Kozhikode, India

## Abstract

*Chromobacterium violaceum* is a gram negative oxidase positive bacillus that causes human infections infrequently. It is a normal inhabitant of soil and stagnant water of the tropical and subtropical areas. In humans, it can cause infections ranging from life threatening sepsis with metastatic abscesses to skin infections and urinary tract infections. The organism is notoriously resistant to most cephalosporins and Ampicillin. Fluoroquinolones and aminoglycosides show good in vitro susceptibility. High mortality rates associated with these infections necessitate prompt diagnosis and appropriate antimicrobial therapy. Here we present three cases of* Chromobacterium violaceum* infection from Government Medical College Kozhikode, Kerala.

## 1. Introduction

*Chromobacterium violaceum* is a gram negative, motile, oxidase positive bacillus that is temperature sensitive and widely distributed in natural aquatic environments. It grows easily on ordinary media like blood agar, MacConkey agar, and nutrient agar producing a violet antioxidant pigment known as violacein [1]. Human infections with this organism, although rare, can result in severe systemic infection by entering the bloodstream via an open wound [2]. Rapid progression to sepsis with metastatic abscesses and multidrug resistance are striking features of* Chromobacterium violaceum* infections. The microorganism, previously thought to be confined to the geographic area between latitudes 35°N and 35°S, may be expanding its habitat beyond this range due to the effects of global warming [1]. Interestingly the monobactam Aztreonam was first described as a natural metabolic product of this bacterium [3, 4].

## 2. Case  1 (Septicaemia)

An 11-month-old male child was referred to our hospital with complaints of high grade fever of 5-day duration, loose stools, and respiratory distress. The fever was preceded by cellulitis of the right cheek and cervical and preauricular adenitis. He was injected with Cefotaxime at the time of referral. On admission the child was febrile with pallor, cervical lymph node enlargement, and hepatosplenomegaly. Blood work-up (Table 1) showed anaemia. Peripheral smear examination reported severe microcytic hypochromic anaemia with neutropenia. Smear for malaria parasite examination was negative. X-ray examination showed multiple patchy opacities in the lungs. A provisional diagnosis of bronchopneumonia with lymphoreticular malignancy was made and the child was empirically put on Cefotaxime injection of 350 mg IV Q8H, Ampicillin injection of 350 mg IV Q6H, Vancomycin injection of 140 mg IV Q18H, and Oseltamivir of 30 mg oral BD along with other supportive measures. Second day after admission, the patient's condition worsened and he was given transfusions of fresh frozen plasma and packed cells. In spite of intensive treatment, the patient succumbed to death 48 hours after admission.

**Figure 1:**
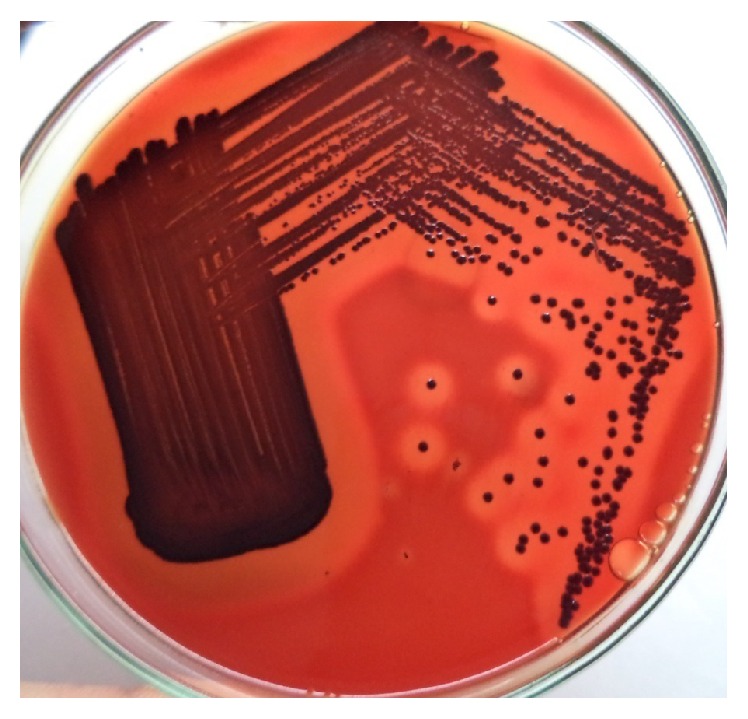
*β*-Hemolytic colonies on blood agar.

**Figure 2:**
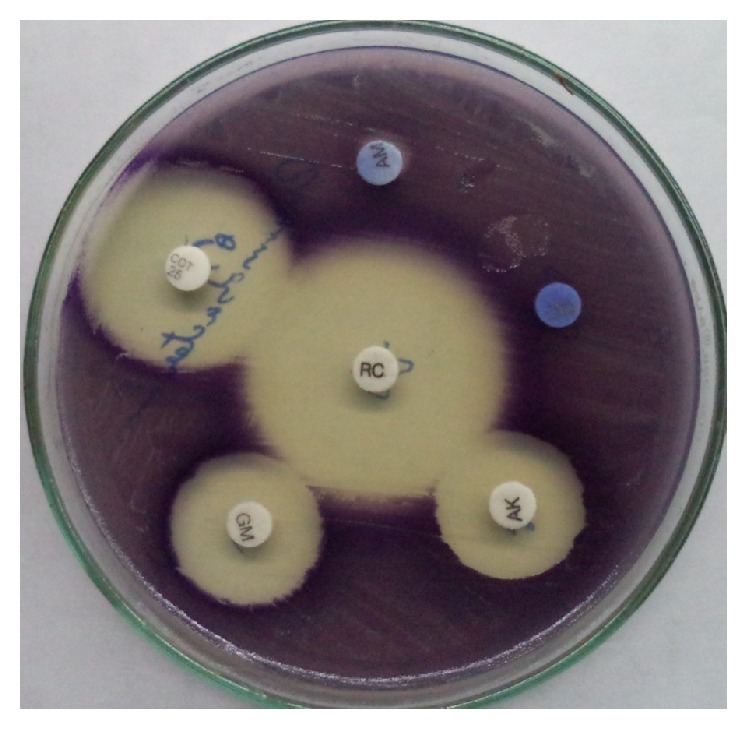
Kirby Bauer disc diffusion method.

**Figure 3:**
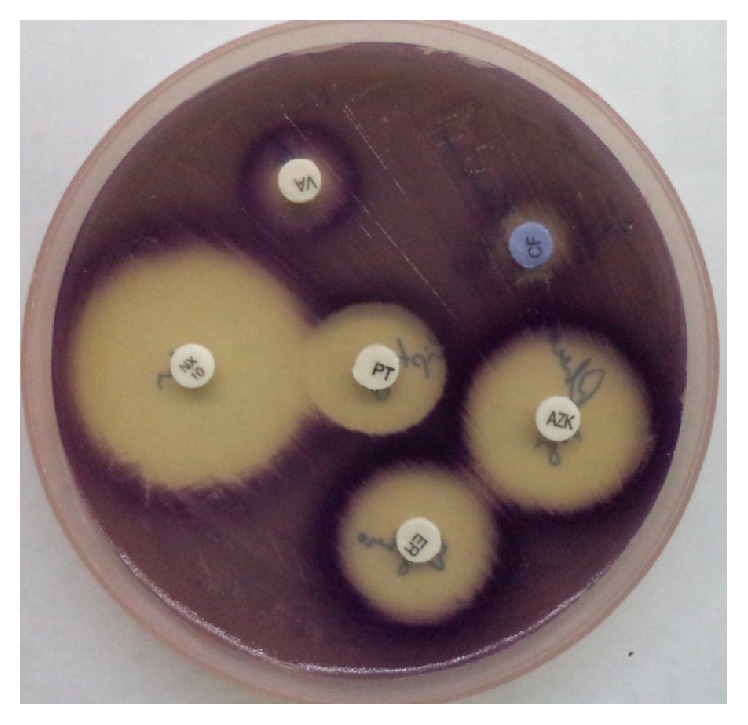
Kirby Bauer disc diffusion method.

A blood culture was sent at admission in brain heart infusion broth which was incubated at 37°C. Subcultures were done on blood agar, on MacConkey agar, and subsequently on nutrient agar which demonstrated numerous colonies with dark violet pigmentation (Figure 1). The organism was gram negative, motile, catalase positive, and oxidase positive. Testing of oxidase reaction by the popular method of Kovacs where the bacterial growth is smeared onto a filter paper impregnated with 1% aqueous solution of tetra methyl p-phenylene diamine dihydrochloride presented with a problem since the organism had violet pigmentation. Hence oxidase reaction was tested by the method described by Dhar and Johnson [5, 6]. The organism was identified as* Chromobacterium violaceum* based on biochemical characteristics and pigment production. It was further confirmed by Vitek-2 system Version: 07.01 (BioMerieux, France) using gram negative card. Antibiogram was done by Kirby Bauer's disk diffusion susceptibility testing technique (Figures 2 and 3) and minimal inhibitory concentration (MIC) method. The results were interpreted as per the Clinical and Laboratory Standards Institute (CLSI) guidelines for other non-Enterobacteriaceae [7]. As the isolate was resistant to Ampicillin and intermediate sensitive to Cefotaxime (Table 2), before getting the proper antibiotic treatment, patient condition deteriorated and developed fatal septicaemia.

## 3. Case  2

A 2.5-year-old male child presented with painful swelling of the scalp and fever of 1-week duration. He had Kawasaki disease at the age of 7 months, measles at the age of 1.5 years, and recurrent episodes of loose stools over the past 1 month. Ultrasound examination of the scalp swelling reported it as “pyemic abscess over the scalp with underlying invasion of both parietal bones extending to extradural space through anterior fontanelle.” Patient was initially treated with oral amoxicillin + Clavulanic acid and later changed to Ampicillin injection and cloxacillin injection.

Blood culture sample was sent in brain heart infusion broth to the laboratory which was incubated at 37°C. Then by subculture on blood agar, on MacConkey agar, and subsequently on nutrient agar, dark violet coloured colonies were grown on all three plates after overnight incubation at 37°C. The organism was biochemically identified as* Chromobacterium violaceum*. Antimicrobial susceptibility testing showed resistance to Ampicillin and susceptibility to fluoroquinolones and aminoglycosides. Cerebrospinal fluid culture did not yield any growth.

The patient progressed to respiratory distress, hypotension, and shock and finally expired within 48 hours of admission before the results of antibiotic susceptibility testing came through.

## 4. Case  3

A 12-year-old school girl presented to the outpatient department with history of intermittent dysuria with fever and chills of 1-week duration. There was no history of any other concurrent illness. Patient gave a history of swimming in pond occasionally [8]. For the last three years, she had recurrent episodes of urinary tract infection. A routine urine examination showed 10–12 pus cells per high power field along with bacteria. Ultrasound examination of the abdomen revealed mild wall thickening of urinary bladder with internal echoes. Routine blood examination was within normal limits.

A mid-stream urine sample was obtained for culture in sterile bottle after following standard precautions and was inoculated on blood agar and MacConkey agar. After overnight aerobic incubation at 37°C, dark violet coloured colonies were observed on blood agar. The biochemical test characteristics were consistent with identification of* Chromobacterium violaceum*. The isolate was resistant to Ampicillin and cephalosporins and sensitive to fluoroquinolones and aminoglycosides.

The patient was empirically started on oral cefixime 200 mg BD. We received one more urine sample for culture after 5 days at the time of review, which yielded the same organism with similar antibiotic sensitivity pattern. The result was again informed to the clinician and the importance of changing the antibiotic to fluoroquinolone was stressed. Subsequently the patient was given oral Ciprofloxacin of 500 mg BD for 7 days. A third urine culture performed at the next hospital visit a week later did not detect any bacteriuria.

## 5. Discussion

The scarcity of reports of human infections with* Chromobacterium violaceum* is astounding given the described ease at which the bacterium is recovered from soil and stagnant water bodies in the tropics and subtropics. The organism has a growth preference for temperatures between 20°C and 37°C. Moist soil and stagnant or slow-flowing water have been the most commonly reported sources of infection, especially in patients who have had cutaneous injury or trauma, which presumably provides a portal of entry for this pathogen. There is no age or gender predilection reported in literature and the only established predisposing disease process has been chronic granulomatous disease [3]. Identification of this organism depends primarily on the biochemical characteristics. A method of detection using multiplex polymerase chain reaction has been described by Scholz and colleagues which is yet largely confined to the realm of research and is not commercially available [1, 9].

The clinical manifestations of* C. violaceum* infections are protean. It has been associated with pneumonia, gastrointestinal tract infections, urinary tract infections, localised cutaneous lesions, localised or metastatic abscesses, osteomyelitis, meningitis, peritonitis, brain abscess, endocarditis, hemophagocytic syndrome, respiratory distress syndrome, and fulminant sepsis [3, 6, 10–12]. The genome of this bacterium has recently been completely sequenced providing a platform for detailed studies of its antiviral and bactericidal activities, cytotoxicity, and drug resistance mechanisms. The virulent strains of* C. violaceum* have elevated levels of superoxide dismutase and catalase that may protect the microorganism from phagocytic attack in humans. This might explain its pathogenicity and fatality in human infections [1, 11]. Pigment production is not a marker of pathogenicity as nonpigmented strains have also been reported to cause infections [12].

Data on antimicrobial susceptibility patterns of* Chromobacterium violaceum* is very limited owing to the rarity of isolation from clinical specimens. Most strains show resistance to penicillins and other beta-lactam antibiotics and, indeed, increased level of beta-lactamase activity has been reported in this organism [1, 11, 13]. Ciprofloxacin is the most effective antibiotic in vitro. It is also susceptible to Gentamicin and Amikacin [1, 3, 10, 11]. In all three cases described above, the patients were primarily on beta-lactam antimicrobials which explains the case fatalities in the first two. In the case of the urinary tract infection, the antibiotic was changed from 3rd generation cephalosporin to Ciprofloxacin only after the second positive culture report. The patient became asymptomatic following the change of antibiotic.

Hence in the tropics and subtropics, infection with* Chromobacterium violaceum* should be one of the differential diagnoses in sepsis, especially if it is preceded by a skin infection or cellulitis. Also, it can present as milder infections like UTI as described in the third case. The inherent resistance pattern of this organism should be borne in mind while instituting empirical antibiotic therapy.

## 6. Conclusion

*Chromobacterium violaceum* is easily isolated from natural aquatic environments of the tropics and subtropics. The traditional geographic distribution pattern of this organism is bound to change in view of the changing global climatic conditions. Human infections with this pathogen, though rare, often result in high mortality rate. Rapid diagnosis and the use of optimal antimicrobials for treatment could be life-saving. Commercial introduction of a cost effective, rapid diagnostic method is the need of the hour. The lack of awareness among clinicians regarding the pathogenesis and antimicrobial resistance pattern of this bacterium is a challenge to be tackled.

## Figures and Tables

**Table 1:** Results of blood tests for Case  1.

Blood test	Results	Reference range/level
Hemoglobin (gm/dl)	5.5	11–14
Platelet count (cell/mm^3^)	65,000	2,00,000–5,00,000
White cell count (cell/mm^3^)	2400	11,000 ± 5000
Neutrophils (%)	25.7	13–33
MCV (fl)	59	78 ± 6
MCH (pg)	18.9	27 ± 2
MCHC (gm/dl)	32.2	34 ± 2
ESR (mm/hour)	60	10

MCV, mean corpuscular volume; MCH, mean corpuscular hemoglobin; MCHC, mean corpuscular hemoglobin concentration; fl, femtoliter; pg, picogram.

**Table 2:** Antimicrobial susceptibility for Case  1.

Antimicrobial	MIC values	Interpretation^*∗*^
Ampicillin	≥32	Resistant
Amoxicillin/Clavulanic acid	≥32	Resistant
Piperacillin/tazobactam	≥128	Resistant
Cefuroxime	≥64	Resistant
Cefotaxime	32	Intermediate
Cefoperazone/sulbactam	32	Intermediate
Ciprofloxacin	≤0.25	Sensitive
Nalidixic acid	≤2	Sensitive
Gentamicin	≤1	Sensitive
Amikacin	≤2	Sensitive
Nitrofurantoin	≤16	Sensitive
Tigecycline	≤0.5	Sensitive
Cotrimoxazole	≤20	Sensitive
Imipenem	≥16	Resistant
Meropenem	≥16	Resistant
Colistin	≥16	Resistant

^*∗*^For other non-Enterobacteriaceae disc diffusion testing is not currently recommended by CLSI. Hence MIC method was used for the interpretation of antimicrobial sensitivity.
